# Data from the experiment of dynamic moisture transport in spruce wood under cyclic step-changes in relative humidity 72–95 %

**DOI:** 10.1016/j.dib.2024.110729

**Published:** 2024-07-17

**Authors:** Jan Richter, Kamil Staněk, Pavel Kopecký

**Affiliations:** a Czech Technical University in Prague, University Centre for Energy Efficient Buildings, Třinecká 1024, Buštěhrad 273 43, Czechia; b Czech Technical University in Prague, Faculty of Civil Engineering, Department of Building Structures, Thákurova 7, Prague 166 29, Czechia

**Keywords:** Data, Experiment, Spruce wood, Dynamic moisture transport, Hygroscopic range, Isothermal conditions

## Abstract

The interaction of wood and moisture has to be considered in many industrial sectors. Wood is highly hygroscopic material while the absorbed moisture affects all its technical properties. One of them is a moisture permeability which is further affected by the sorption hysteresis and also differs in the three wood anatomical directions – radial, tangential, and axial. For the prediction of the dynamic hygro-thermal behaviour of wood can be used numerical simulation tools. However, data from carefully designed and controlled experiments are needed for reliable validation of these tools. This paper presents data from a 45-day dynamic laboratory experiment. The one-dimensional moisture transport in spruce wood in the tangential and radial directions under isothermal conditions was studied. The samples were exposed to cyclic step-changes in relative humidity 72–95 % at 23 °C. Data show the rate of stabilisation of moisture content in the samples, the effect of sorption hysteresis, and changes in the temperature of samples due to moisture sorption. In addition, the paper also presents material functions describing the sorption properties and moisture permeability of spruce wood. These properties were determined based on laboratory measurements using the spruce wood of the same origin as used for the dynamic experiment. The dynamic data, together with the proposed material functions can be used in the development or verification of hygro-thermal numerical simulation tools.

Specifications Table

**Table:** 

Subject	Engineering – Civil and Structural Engineering, Materials – Materials Science Engineering
Specific subject area	Dynamic moisture transport in spruce wood, numerical models of heat and moisture transport
Type of data	Table, Image, Chart, Figure Analysed, Processed
Data collection	For the dynamic experiment was built a new PMMA chamber maintaining 23 °C and allowing an automatic switch in relative between 72 % and 95 %. The masses of the wood samples during the experiment were acquired by manual weighting on the precision balance Sartorius L420S. Prior to each weighing, the balance was set to automatic recording to a MS Excel using software QTREE-DC/RS232 [3]. The temperature and relative humidity inside the chamber were monitored with Rotronic HC2–S sensors and the temperature at the bottom surface of selected samples by type T thermocouples (RS Components Sp. z o.o.). The data were collected in a one-minute interval using dataTaker DT85 logger (Thermo Fisher Scientific Inc.).
Data source location	Czech Technical University in Prague, University Centre for Energy Efficient Buildings, Třinecká 1024, Buštěhrad 273 43, Czech Republic
Data accessibility	Repository name: Mendeley Data Data identification number: doi: 10.17632/dbfdm6sy6x.1 Direct URL to data: https://data.mendeley.com/datasets/dbfdm6sy6x/1
Related research article	J. Richter, K. Staněk, P. Kopecký, P. Schůtová, J. Tywoniak, Dynamic moisture transport in spruce wood – Experiment in hygroscopic range under isothermal conditions, AIP Conference Proceedings, 2894 (2023). https://doi.org/10.1063/5.0164321

## 1 Value of the Data

• Data can improve understanding of dynamic moisture transport in wood.
• Data can be used in the development and verification of numerical simulation tools of moisture transport in wood.
• Data can be beneficial for scientists especially in the field of building physics and material science.
• The detailed description of the experiment can be helpful in developing new experiments, especially in terms of sample dimensions and time periods between weighing.

## 2 Background

The purpose of the measurement was to generate an accurate dataset that describes the dynamic hygrothermal behaviour of spruce wood under well-defined initial and boundary conditions. In the related article [1], charts of the measured data and a brief description of the experiment were presented. This data article provides a full description of the samples and the experimental method that makes it reproducible. It also provides a dataset of measured values together with boundary conditions. In addition, the data file includes functions that describe the basic properties of the wood used and that can be easily applied to hygrothermal numerical tools. These functions were determined based on laboratory measurements using wood of the same origin as for samples used in the dynamic experiment.

## 3 Data Description

The dataset is available as an MS Excel file accessible in the Mendeley Data repository [2]. The file contains measured data from a dynamic hygrothermal experiment with spruce wood at a constant temperature of 23 °C and cyclic changes in ambient relative humidity between 72 % and 95 %. The file contains four numbered sheets and two appendix sheets. The numbered sheets include: (1) the basic data about the samples, (2) the data of mass and moisture content variations of the samples during the experiment, (3) the data of the temperature changes in the samples caused by moisture absorption/desorption, and (4) boundary conditions during the experiment. The two appendix sheets provide material functions that are essential for the use of the data for the verification of hygrothermal numerical tools: (Appendix A) hygroscopic sorption properties and (Appendix B) moisture permeability. They were measured on wood of the same origin as for the samples used in the dynamic experiment.

The description of data can be found in Table 1.

**Table 1:** Content of the related dataset [2].

Sheet no.	Title	Content
1	Samples and specimens	Basic data of samples and wood specimens – dry mass, dimensions, volume, and bulk density.
2	Mass and moisture content	Measured masses and calculated moisture content of wood samples during the experiment.
3	Temperature difference	Measured temperature difference between the bottom surface of the selected sample and the ambient air.
4	Boundary conditions	Boundary conditions of the experiment. *T*_amb_ – measured temperature [°C] of the air in the wind tunnel (average from four sensors – at the beginning and at the end of both wind tunnels) *φ*_amb_ – measured relative humidity [–] of the air in the wind tunnel (average from four sensors – at the beginning and at the end of both wind tunnels) *β* – mass transfer coefficient [m/s] on the exposed upper surface of the wood specimens determined by a separate experiment described in section see section “Surface mass transfer coefficients”. *α*_c_ – convective heat transfer coefficient [W/(m^2^K)] on the exposed upper surface of the wood specimens that was calculated using a simplified expression of the Lewis formula [4]. It is included in the dataset as an additional information for the purpose of verification of hygrothermal numerical tools. *α*_r_ – radiative heat transfer coefficient [W/(m^2^K)] on the exposed upper surface of the wood specimens that was calculated based on Stefan-Boltzman law.
Appendix A	Sorption properties	Expression and fitting parameters describing the main absorption and desorption isotherms determined for spruce wood of the same origin as used in the dynamic experiment. The measurement method and preliminary functions were firstly published in [5]. The sheet includes also measured data describing hysteretic behavior of the same wood and fitting parameters of the suggested hysteresis model are given in [6].
Appendix B	Moisture permeability	Expressions and fitting parameters describing the water vapour diffusion resistance factor of spruce wood as a function of relative humidity and, alternatively, moisture content. Their validity is limited to relative humidity higher than 70 % or the corresponding moisture content range. The measurement method and measured data were firstly published in [7]. Again, the measurement was performed with wood of the same origin as used in the dynamic experiment.

## 4 Experimental Design, Materials and Methods

### 4.1 Apparatus

**Fig. 1:**
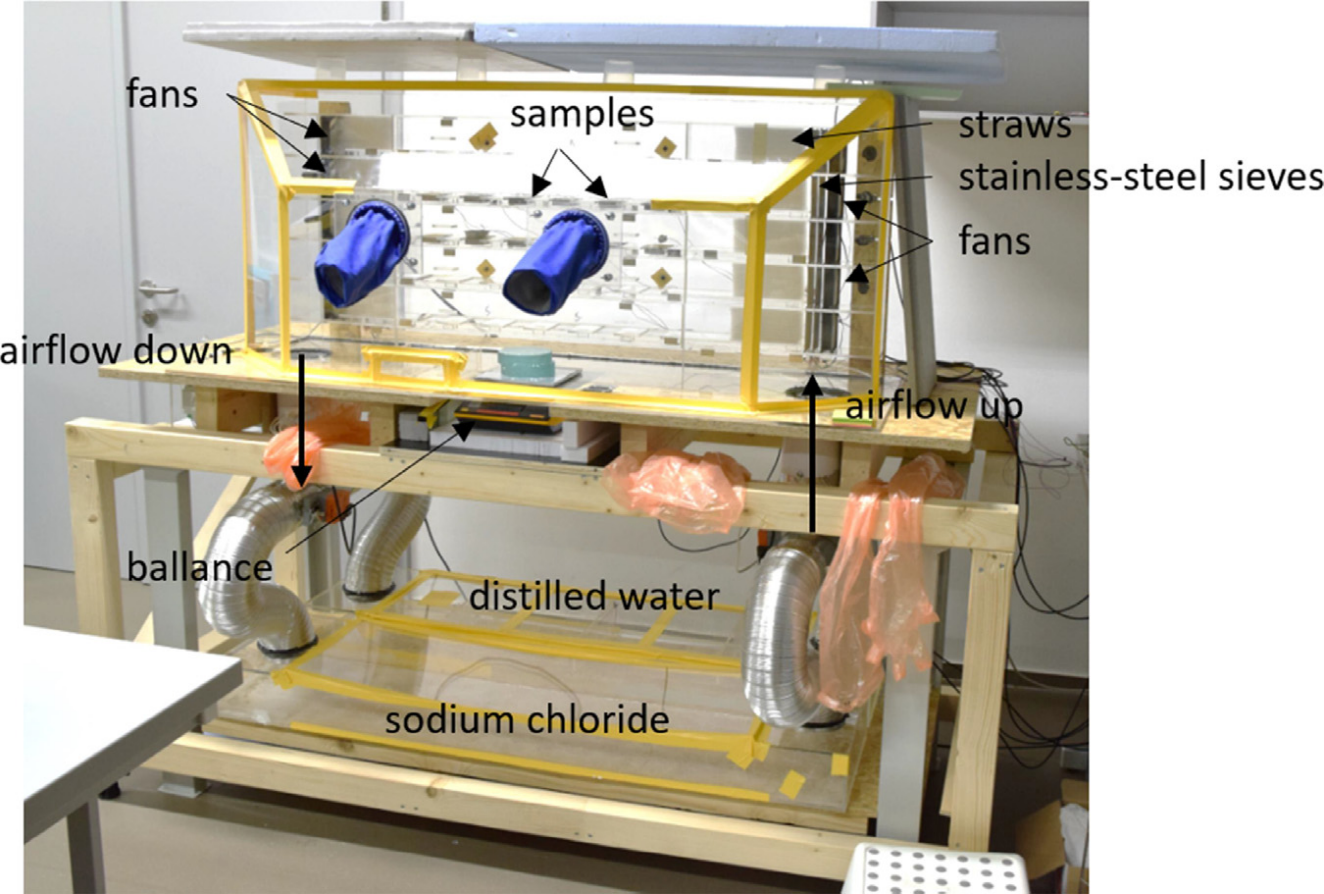
Experimental apparatus. (Figure was firstly published in [1]).

A new apparatus was built for the experiment, see Fig. 1. Its main chamber was made out of 6 mm PMMA. The chamber was connected to the two vessels below it by aluminium piping. Two opposite-orientated fans were mounted in the piping to circulate air between the chamber and the vessels. Two flaps on both sides of the piping were used to control which vessel exchanges the air with the main chamber. During the experiment, one vessel contained distilled water, and the other a saturated solution of sodium chloride. When the flap position changed, a step-change in relative humidity was achieved in the main chamber. The vessel containing distilled water was the source of 100 % relative humidity, and the container of saturated sodium chloride solution was the source of 75 % relative humidity, at 23 °C [8]. As a result of the heat generated by the fans and the natural upward movement of the warmer air, the temperature in the main chamber was kept approximately 2 °C higher than the temperature in the vessels. Therefore, the relative humidity in the main chamber was approximately 95 % and 72 %, respectively. The flaps enabled automated switching at selected time intervals.

**Fig. 2:**
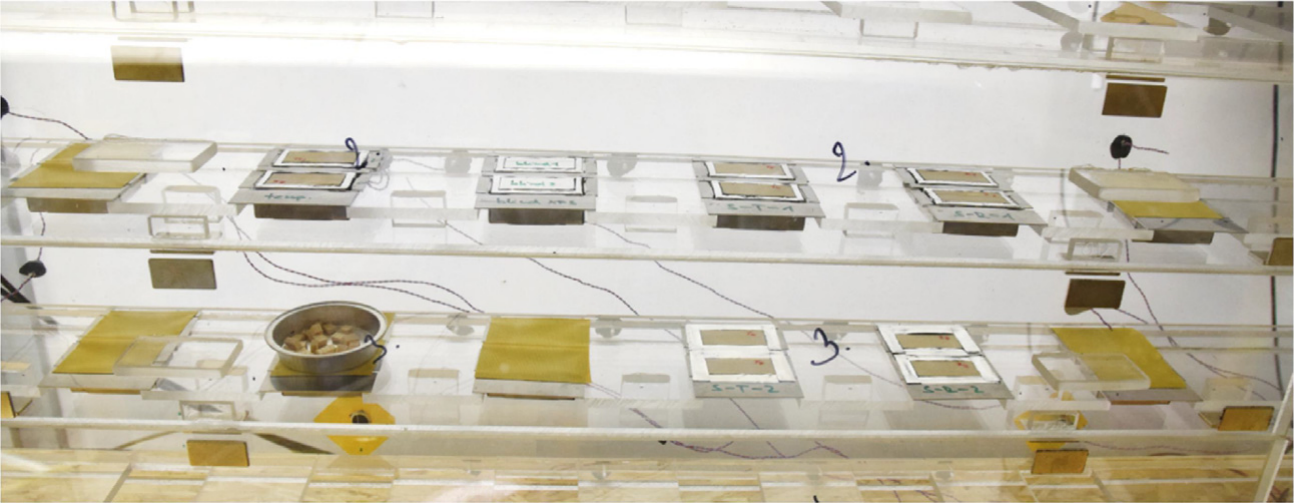
Samples placed in two wind tunnels: top right to left - SR1, ST1, blind, temperature sample; bottom right to left – SR2, ST2, control sample. (Figure was firstly published in [1]).

In the back of the main chamber there were five small wind tunnels with a cross section of 92 × 92 mm^2^ with six positions for the samples in each tunnel, see Fig. 1, Fig. 2. Axial fans with adjustable speed were installed at the inlet and outlet of the tunnels. The airflow was directed from right to left in the frontal view of the chamber. A pack of small diameter tubes was installed at the beginning and end of each tunnel to keep airflow above the samples laminar and stable. Each sample was represented by a stainless-steel dish containing two wood specimens, see Fig. 2. The dishes were designed that their upper surfaces were aligned with the upper surfaces of the specimens and also with the tunnel floor to prevent disturbances in the airflow. The mass transfer coefficients on the top surface of the specimens were determined for each measured position prior to the experiment (see section “Surface mass transfer coefficients”). The values were found to be in good mutual agreement in all selected positions.

A pair of gloves was installed in the front wall of the main chamber for handling and weighing the samples without affecting the humidity conditions inside. Each wind tunnel had a removable vertical front cover held in place by magnets. Another cover was placed at the bottom of the main chamber. Below it was the balance which was placed outside the chamber to protect it from the humid environment, see Fig. 1. There was also a concern that vibrations of the whole apparatus caused by numerous fans can affect the accuracy of the balance. Therefore, the balance was placed on a separate support that did not touch the rest of the apparatus.

A small drawer was designed in the front wall of the main chamber to serve as the only option to insert or remove samples or other equipment. It was primarily used to insert conditioned samples at the beginning of the experiment.

Rotronic HC2–S temperature and humidity sensors were used to measure the conditions in the apparatus: at the inlet and outlet of each wind tunnel with samples, in the main chamber near the balance, and inside both vessels with humidistatic media. Another sensor monitored the conditions in the laboratory. All data were recorded in one-minute intervals and collected by the dataTaker DT85 logger.

Before the experiment, the chamber without samples was conditioned for several weeks under the humidity conditions of the experiment. Because PMMA swells and shrinks under changes of surrounding relative humidity, the walls of the main chamber deformed and some of the joints broke. Flexible airtight tape was used for the repair, which also allowed for further cyclic deformation during the experiment. The back part of the chamber containing the air tunnels resisted changes in relative humidity without the deformation.

### 4.2 Samples

**Fig. 3:**
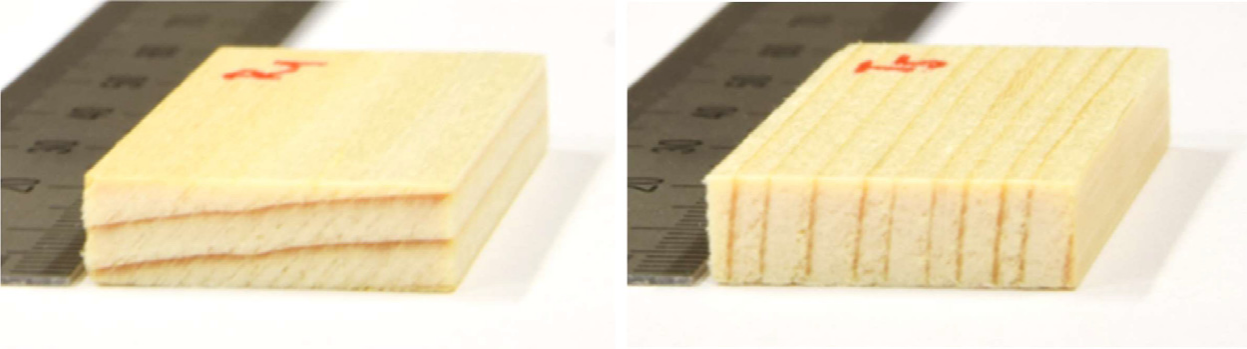
Wood specimens for studying moisture transport in radial (left) and tangential (right) directions. (Figure was firstly published in [1]).

Individual specimens of spruce wood (*Picea Abies*) were cut that their upper surface was perpendicular to either the radial or tangential anatomical direction, see Fig. 3. These specimens were referred to as tangential (T) and radial (R), respectively. The specimens had dimensions of 20 × 50 × 5 mm^3^. Detailed information on each specimen is provided in the data file related to this article [2]. The samples were dried at a temperature 22.5 °C for six weeks using a molecular sieve. The dry bulk density of each specimen was determined and pairs of specimens of similar density were selected for the experiment – four tangential and four radial.

**Fig. 4:**
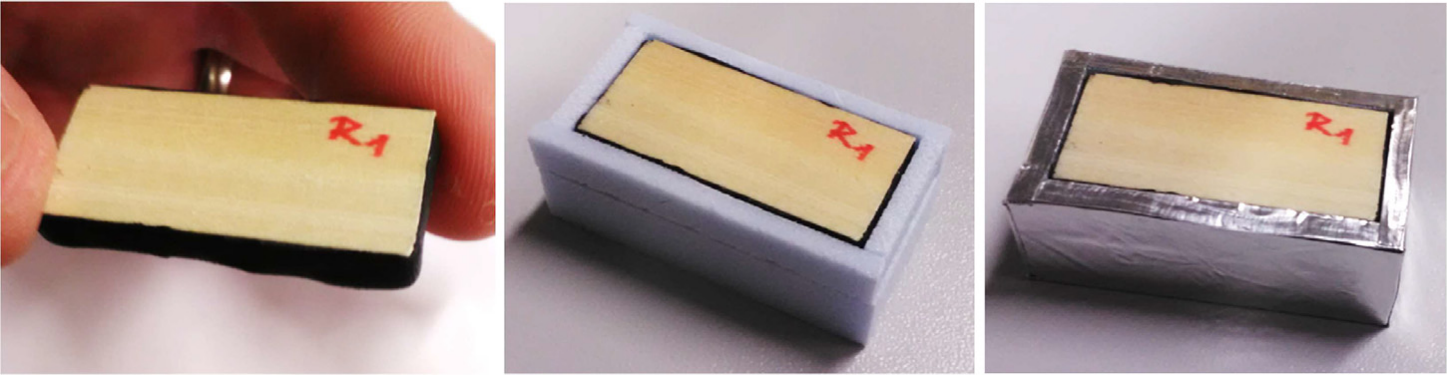
Specimens sealed with vapour-tight tape (left), covered with XPS blocks (middle) and masked with aluminium tape (right). (Figure was firstly published in [1]).

**Fig. 5:**
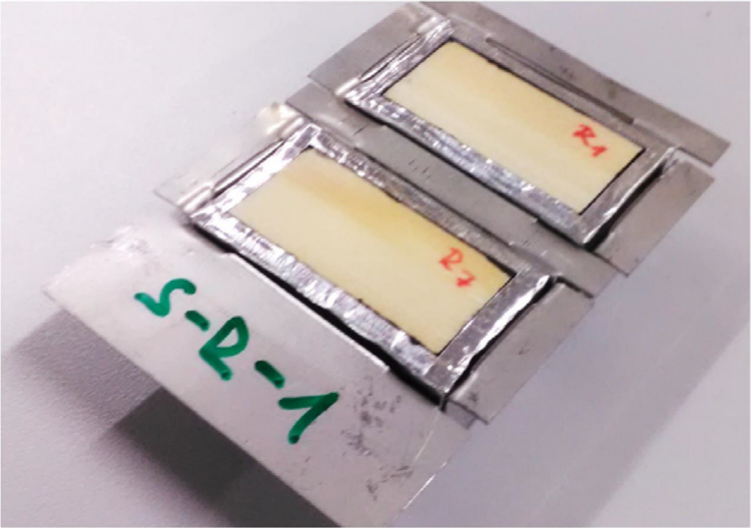
Weighed sample with radial specimens. (Figure was firstly published in [1]).

The dried specimens were sealed on five sides with vapour- and air-tight butyl tape, leaving only the top surface open to the ambient environment, see Fig. 4-left. The specimens were then insulated with extruded polystyrene with a thickness of 3 mm on the sides and 8 mm on the bottom, see Fig. 4-middle. Finally, the specimens were sealed with aluminum tape, see Fig. 4-right. The aim of the sample wrapping was to create an adiabatic and impermeable boundary on all sample surfaces except the exposed one and to achieve one-dimensional one-sided heat and moisture transport. All pieces of all materials used for the completion of samples were weighed before and after the experiment. Pars of specimens were embedded in stainless-steel dishes (see Fig. 5). The dish with specimens represented a single sample which was weighed during the experiment. In the experiment two samples (dishes) with radial specimens and two with tangential specimens were used, labelled S-R (spruce–radial) and S-T (spruce–tangential), respectively.

**Fig. 6:**
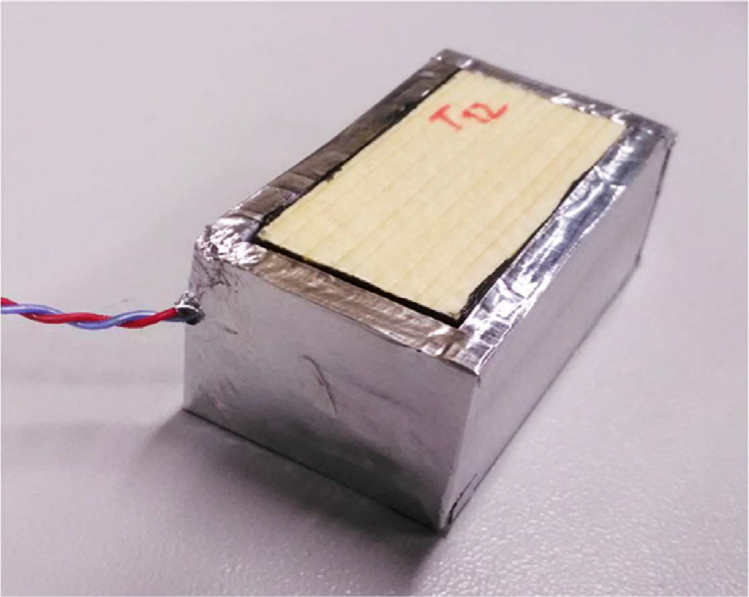
Wooden specimen equipped with a temperature sensor at the bottom surface. (Figure was firstly published in [1]).

A single extra dish was used to measure temperature variations at the thermally-insulated bottom surfaces of the specimens. This dish was not weighed during the experiment. It consisted of one radial and one tangential specimen and temperature sensors (type T thermocouple) placed between the wood and the butyl tape in the centre of the bottom surfaces, see Fig. 6.

**Fig. 7:**
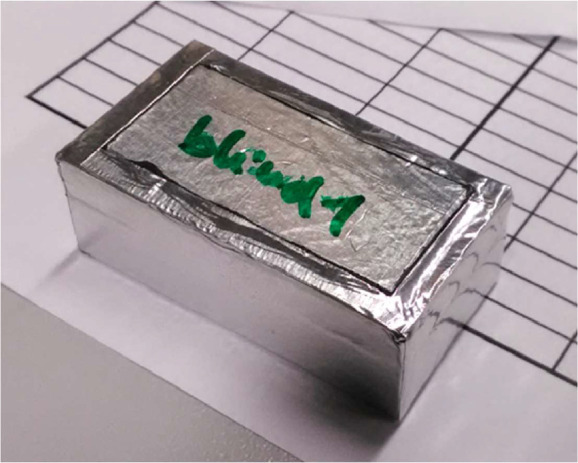
Blind sample with non-hygroscopic specimens. (Figure was firstly published in [1]).

An additional sample, referred to as the “blind sample”, was used for the correction of the measured masses. It consisted of a dish containing two non-hygroscopic specimens, see Fig. 7. By weighing it together with the other samples, it was possible to capture the mass variations due to small amounts of adsorbed and desorbed moisture on other non-wooden parts of the sample assemblies under changes in ambient relative humidity. The mass variations were used as a correction for weiged masses of the other tested samples.

Finally, an aluminum dish containing wood splinters was used to capture the equilibrium moisture content under each change in the boundary conditions, see Fig. 2-bottom left. This dish is in the data file referred to as the “reference sample”.

### 4.3 Conditioning

After drying the samples at 0 % relative humidity and their completion in the laboratory at about 40 % relative humidity, they were placed inside a desiccator containing a saturated solution of sodium chloride. Samples were periodically weighed until an equilibrium moisture content was reached. This procedure ensured that the initial equilibrium moisture content of the samples was at 75 % relative humidity on the main absorption isotherm.

### 4.4 Procedure

The main chamber of the experimental apparatus was set to maintain a relative humidity of 72 % at a temperature of 23 °C for one week. Meanwhile, the samples were conditioned at a relative humidity of 75 % and a temperature of 22.5 °C. The samples were then inserted into the main chamber using the drawer in the front wall, weighed and left for 24 h for their relaxation. After that, the samples were weighed and the test started.

The whole test procedure was divided into two parts. In the first part, the samples were exposed to cyclic step changes in relative humidity of 95/72 % at 23 °C, while each of the two conditions was maintained until the equilibrium moisture content of the samples was reached. In total, three cycles were performed. Then, it was intended to study the moisture transport under lower airflow speed above samples. The initial speed measured 10 mm above samples of 1.0 m/s was changed to 0.3 m/s. However, during the fourth cycle, instead of studying the effect of wind speed, it was revealed that one of the main fans in the piping collapsed and the ambient relative humidity during the fourth cycle deviated from the intended value. It is also reflected in the weighed masses of the specimens. However, ambient conditions were still recorded, therefore, the data of this fourth cycle are still representative for the hygric behaviour of samples. The equilibrium moisture content of the specimens after each change in the boundary conditions was reached after ca. 4–5 days. The first part of the experiment (four cycles) took 42 days.

In the second part of the experiment, the time intervals for switching the humidity conditions were set to 16/8 h for humidity levels of 95/72 %, respectively. The airflow speed was still kept at 0.3 m/s. Three full cycles were performed under these conditions. The second part of the experiment lasted 3 days.

### 4.5 Weighing on balance

Although the weighing was performed on balance outside the chamber, there was a shield around the balance that reduced convection around weighed samples. Each sample was exposed to laboratory conditions during weighing for 30–40 s.

All masses were hand-weighed. Prior to each weighing the balance was set to an automatic recording in 10-second interval into MS Excel sheet. For this purpose, software QTREE-DC/RS232 was used [3].

### 4.6 Data processing and evaluation

The difference (Δ*m*_b_) between the actual and initial mass of the blind sample at each weighing was calculated using Eq. (1). This difference was subtracted from the mass of the whole sample (*m*_s_) together with the mass of non-wooden parts of the dish assembly (*m*_n_): steel dish, butyl sealant, aluminum tape, and initial mass of extruded polystyrene. In this way, masses of moist wood (*m*_w_) were obtained, see Eq. (2).

(1) $$\Delta m_{b\left(i\right)}=m_{b\left(i\right)}-\;\Delta m_{b\left(1\right)}$$

where:

(2) $$\begin{array}{ccc} & & \Delta m_{b\left(i\right)}-\text{difference}\;\text{of}\;\text{mass}\;\text{of}\;\text{the}\;\text{blind}\;\text{sample}\;\text{at}\;\text{time}\;\left(i\right)\;\text{from}\;\text{its}\;\text{initial}\;\text{mass}\;\left[\text{kg}\right]\\ & & m_{b\left(i\right)}-\text{mass}\;\text{of}\;\text{the}\;\text{blind}\;\text{sample}\;\text{at}\;\text{moment}\left(i\right)\;\left[\text{kg}\right]\\ & & m_{b\left(1\right)}-\text{first}\;\text{weighed}\;\text{mass}\;\text{of}\;\text{the}\;\text{blind}\;\text{sample}\;\text{during}\;\text{the}\;\text{experiment}\;\left[\text{kg}\right]\\ & & m_{w\left(i\right)}=m_{s\left(i\right)}-\;\Delta m_{b\left(i\right)}-m_{n\;} \end{array}$$

where:

$$\begin{array}{ccc} & & m_{w,\left(i\right)}-\text{mass}\;\text{of}\;\text{wet}\;\text{wood}\;\text{at}\;\text{moment}\left(i\right)\;\left[\text{kg}\right]\\ & & m_{s,\;\left(i\right)}-\text{mass}\;\text{of}\;\text{the}\;\text{sample}\;\left(\text{the}\;\text{assembled}\;\text{dish}\right)\;\text{at}\;\text{moment}\left(i\right)\;\left[\text{kg}\right]\\ & & m_{n\;}-\text{mass}\;\text{of}\;\text{non}\;\text{-}\;\text{wooden}\;\text{parts}\;\text{of}\;\text{the}\;\text{sample}\;\left[\text{kg}\right] \end{array}$$

Moisture content (*u*) at each weighing was determined based on the following equation:

(3) $$u_{\left(i\right)}=\frac{m_{w\left(i\right)}-m_{d\left(i\right)}}{m_{d\left(i\right)}}$$

where:

$$\begin{array}{ccc} & & u_{\left(i\right)}-\text{moisture}\;\text{content}\;\text{at}\;\text{moment}\;\left(i\right)\;\left[\text{kg}/\text{kg}\right]\\ & & m_{d\left(i\right)}-\text{mass}\;\text{of}\;\text{dry}\;\text{wood}\;\text{at}\;\text{moment}\left(i\right)\;\left[\text{kg}\right] \end{array}$$

The data of the ambient temperature and relative humidity above the samples were averaged from the sensors in the inlet and outlet of both wind tunnels with samples (average of four sensors).

To calculate the temperature differences between the air above the samples and the insulated bottom surface of the samples, the two values were subtracted. Since a small constant deviation from zero was found under equilibrium conditions, the data was corrected using a constant offset.

### 4.7 Surface mass transfer coefficients

As the samples were only five millimeters thick and having quite high moisture permeability, it was important to determine the surface mass transfer coefficient for reliable use of the data in terms of validation of numerical tools. For this purpose, the following experiment was performed. Steel dishes were filled with distilled water to the brim and placed in different positions in the wind tunnels under an ambient relative humidity of 72%. The loss in their mass over time due to evaporation was recorded. The measurement was repeated twice with very consistent results. The mass transfer coefficient was calculated based on the first Fick's law. As the values differ only up to 15 % for the different positions in the wind tunnels, only the average is given in the data file. The values 0.0135 and 0.0070 m/s of the mass transfer coefficient were used for wind speeds of 1.0 and 0.3 m/s, respectively.

### 4.8 Uncertainties

Calculation of the mass of wood specimens during each weighing required subtractions of several other weighed masses: the mass variations of the blind sample and the constant mass of the non-wooden parts of the dish assemblies. The standard uncertainty of the balance in this load range is ± 1.7 mg. It means that, recalling the error propagation rule, the standard uncertainty of any given mass of wood specimens can be estimated as ± 3.4 mg, while the expanded uncertainty as ± 7 mg, for approximately 95 % confidence interval.

The expanded uncertainty of the moisture content values can be estimated as ± 0.008 kg/kg, which is around ± 4 % relative to the values presented.

## Limitations

• Weighing on balance was performed outside the chamber. Although, there was a shield around the balance that reduced convection around weighed samples, each sample was exposed to conditions in the laboratory during weighing for 30–40 s.
• The measured changes in the mass of the blind sample were quite high. It confirms the importance of this sample. If reproducing the experiment, there can be more than one blind sample or more different variants of it for more confidential correction of the data.
• Weighing was not performed at hour 1032. Therefore, the masses of the samples at this time are missing.

## Ethics Statement

The authors confirm that they have read and follow the ethical requirements for publication in Data in Brief and confirm that the current work does not involve human subjects, animal experiments, or any data collected from social media platforms.

## CRediT authorship contribution statement

**Jan Richter:** Methodology, Investigation, Data curation, Writing – original draft. **Kamil Staněk:** Conceptualization, Methodology, Data curation, Writing – review & editing. **Pavel Kopecký:** Conceptualization, Methodology, Writing – review & editing.

## Data Availability

Experimental data_2021_Dynamic moisture transport in wood (Original data) (Mendeley Data).
